# Impact of osteoporosis pharmacotherapy and vitamin d supplementation on fracture morphology and treatment of pelvic fragility fractures: a retrospective cohort study of 1493 patients from the German Pelvic Trauma Registry

**DOI:** 10.1007/s11657-026-01687-9

**Published:** 2026-03-16

**Authors:** Alexander Trulson, Stefan Huber, Eftychios Bolierakis, Till Berk, Fabian Stuby, Andreas Höch, Christian Kleber, Isabel Graul, Philipp Pieroh

**Affiliations:** 1 Department for Traumatology and General Surgery, BG Trauma Center Murnau, Murnau, Germany; 2 AUC-Academy for Trauma Surgery, Munich, Germany; 3 Department of Orthopaedics, Trauma and Reconstructive Surgery, Medical Faculty, RWTH Aachen University, Aachen, Germany; 4 Department of Orthopedic, Trauma and Plastic Surgery, University of Leipzig, Leipzig, Germany; 5 Eberhard-Karls-University, Tübingen, Germany; 6 Department of Trauma-, Hand and Reconstructive Surgery, University of Jena, Jena, Germany; 7 German Pelvic Injury Register of the German Society of Traumatology (Deutsche Gesellschaft fuer Unfallchirurgie [DGU]), Munich, Germany

**Keywords:** Fragility fractures of the pelvis, Pelvic ring injuries, Osteoporosis management, Secondary fracture prevention, Geriatric trauma, Treatment gap analysis

## Abstract

***Summary*:**

Osteoporosis causes bone fragility and fractures, yet most patients receive no treatment. This German registry study of nearly 1500 elderly pelvic fracture patients found that two-thirds were untreated before fracture. Paradoxically, treated patients sustained more severe, displaced fractures requiring surgery more often, possibly reflecting advanced disease severity or altered bone properties from medication.

**Background:**

Fragility fractures of the pelvis (FFP) are related to low-energy trauma and osteoporosis. Currently, data on secondary pharmacological fracture prevention and their effect on fracture morphology is rare. We aimed to determine the proportion of patients receiving specific osteoporosis medication (SOM), vitamin D supplementation only (VitD), or no treatment (NOT) before and after a FFP. Secondarily, we evaluated possible differences in fracture morphology and treatment in the study population.

**Methods:**

This retrospective cohort study analyzed data from the German Pelvic Fracture Registry (2018–2024) of patients ≥ 60 years with an FFP. Patients were grouped hierarchically in the following groups: SOM, VitD, or NOT. Demographics, fracture morphology, treatment, and postfracture group changes regarding osteoporosis treatment were compared between groups.

**Results:**

Of 1493 patients, mean age 82.34 ± 8.06 years, predominantly female (83.5%), 98 (6.6%) had a SOM, 386 (25.9%) VitD, and 1009 (67.6%) NOT before FFP. The SOM group was significantly older, predominantly female (*p* < 0.05), and demonstrated higher prefracture functional independence (71.4% SOM vs. 58.5% VitD vs. 66.4% NOT; *p* = 0.018). FFP Type IV fractures occurred more frequently in the SOM group (33.7% SOM vs. 23.3% VitD, 19.8% NOT; *p* = 0.006) and were more frequently operatively treated (45.9% SOM vs. 30.4% VitD, 29.2% NOT; *p* = 0.006). Following a FFP, in 527 patients (35.9%), the treatment regime was escalated; in 463 patients (31.0%), no change occurred.

**Conclusions:**

Despite established treatment guidelines, two-thirds of patients who sustained an FFP received neither SOM nor VitD in Germany. Patients with prefracture SOM treatment sustained more unstable fractures requiring surgical intervention, possibly reflecting advanced disease severity or altered bone biomechanics. Secondary fracture prevention remains suboptimal, with one-third of patients not receiving treatment postfracture.

## Introduction

Osteoporosis represents a systemic skeletal disorder characterized by reduced bone strength and increased fracture risk. Osteoporotic fractures result in increased morbidity, mortality, functional impairment, healthcare costs, and diminished quality of life [1–4, 15]. With demographic aging, the global incidence is projected to rise substantially [5, 6], establishing osteoporosis as a major public health concern [5, 7–9].

Fragility fractures of the pelvis (FFPs) constitute a distinct category of pelvic injuries that occur following low-energy trauma in patients with compromised bone quality, predominantly due to osteoporosis. Rommens and Hofmann identified characteristic morphological differences between osteoporosis-associated pelvic ring fractures and typical high-energy trauma patterns. Based on these observations, they established the FFP (fragility fractures of the pelvis) classification system specifically designed to categorize fragility fractures of the pelvic ring, which has since been widely adopted as the standard classification tool for this fracture type [34]. The FFP classification categorizes fragility fractures into four progressively severe main patterns (FFP I–IV), with each category further divided into subcategories that characterize the specific injury pattern in detail. FFP I are isolated anterior pelvic ring fractures without posterior involvement, FFP II are nondisplaced posterior ring fractures that maintain stability (FFP II). FFP III are characterized by unilateral displaced posterior ring fractures causing rotational or vertical instability (FFP III), whereas FFP IV are bilateral displaced posterior ring fractures representing complete bilateral instability and the highest degree of structural disruption. The subcategories within each main group enable precise documentation of fracture morphology, displacement patterns, and involvement of specific pelvic structures. Although pelvic fragility fractures are not formally included in the WHO/FRAX definition of “major osteoporotic fractures” [40], they are increasingly recognized in clinical discourse as the “fifth major osteoporotic fracture” or as an “overlooked” osteoporotic fracture entity. Sahota et al. explicitly describe pelvic fragility fractures as a “neglected group of osteoporotic fractures” associated with significant morbidity and increased mortality [41, 42]. The primary therapeutic objective in osteoporosis management involves the prevention of fragility fractures through appropriate pharmacological intervention. While the occurrence of a fragility fracture constitutes sufficient clinical evidence for the diagnosis of osteoporosis [10–12], substantial deficiencies persist in both diagnostic recognition and therapeutic management globally, particularly in the context of secondary fracture prevention [13, 14]. Kendler et al. conducted a comprehensive analysis revealing that among patients presenting to primary care following a fragility fracture, 61.3% remained undiagnosed and 60.2% received no therapeutic intervention for osteoporosis during a mean observation period of 363 days (range 91–808 days) [16]. This therapeutic inertia is particularly concerning given the explicit recommendations of current international and German clinical practice guidelines for postmenopausal osteoporosis [10, 12, 17, 18], which advocate for immediate clinical diagnosis and prompt initiation of pharmacological therapy following any fragility fracture, irrespective of additional risk factors. Contemporary literature demonstrates that the majority of patients sustaining FFPs have not received appropriate osteoporosis evaluation or treatment prior to fracture occurrence.

Several studies have shown that only 14 to 25% of patients received an antiosteoporotic medication prior to the fragility fracture, initiation rates after fractures varied between 4 and 14.1% [23–25]. Also, prefracture osteoporosis treatment demonstrated superior functional recovery, fewer refractures, and higher quality-of-life scores [27].

The relationship between prefracture osteoporosis management and patient functional status remains incompletely characterized.

Current findings on calcium supplementation indicate limited therapeutic benefit. Monotherapy with calcium, either alone or in combination with vitamin D, has no significant effect on fracture prevention in patients without concomitant vitamin D deficiency [19]. Current systematic reviews evaluating the efficacy of established osteoporosis therapies show that SOM (bisphosphonates, denosumab, teriparatide, and raloxifene) significantly reduce the incidence of fragility fractures compared to placebo [20, 21]. However, the impact of specific osteoporotic medications on fracture patterns presents clinical considerations. Bisphosphonates and denosumab, while effective in reducing overall fracture risk through improved bone mineral density, may alter bone metabolism in ways that potentially influence fracture morphology and stability patterns [28]. Black et al. analyzed 196,129 women over 10 years and found that while atypical femur fractures occur rarely (1.74 per 10,000 patient-years), the risk increases substantially with prolonged bisphosphonate use [22]. Given that both femoral and pelvic bones are weight-bearing structures susceptible to stress-related changes under bisphosphonate therapy, similar considerations may apply to osteoporotic pelvic bone behavior. 

Hospital-based osteoporosis treatment initiation represents a critical opportunity for secondary fracture prevention. Treatment gaps persist, with studies reporting that only 4% of treatment-naive patients with pelvic fragility fractures began antiosteoporotic therapy within one year post-fracture, while 92.3% never received such treatment during extended follow-up periods [29].

Despite the clinical significance of FFPs, limited data exist regarding the specific relationship between osteoporosis treatment status and fracture morphology, treatment, and postfracture therapy. Understanding these associations is essential for optimizing both preventive strategies and acute management protocols for this vulnerable patient population.

The present study aimed to determine the prevalence of patients receiving specific osteoporosis pharmacotherapy (SOM), vitamin D supplementation (VitD), or none of them (NOT) both prior to and following an FFP. Secondary objectives included evaluation of potential differences in fracture morphology and treatment approaches among patients receiving different osteoporosis treatment regimens for FFP. We hypothesize that prefracture osteoporosis treatment status influences the clinical presentation and management of fragility fractures of the pelvis.

## Materials and methods

This retrospective cohort study utilized data from the German Pelvic Fracture Registry—a part of the TraumaRegister of the German Trauma Society DGU®—maintained by the DGU and hosted by the Academy for Trauma Surgery Munich, Germany. The German Pelvic Fracture Registry is a well-established trauma registry that has been collecting data for over 30 years. It retrospectively captures data on pelvic ring and acetabular fracture management. Twenty-two centers voluntarily participate in the registry and are evenly distributed across Germany of all federal states. Participating institutions include university hospitals, specialized trauma centers, and smaller hospitals. Data were obtained in accordance with the General Data Protection Regulation (GDPR) and the responsible local ethics committee approved data collection in all participating institutions and on the basis of informed consent of the included patients.

Pseudonymized data of 22 centers was included.

### Patient cohort and inclusion criteria

Our patient selection strategy was designed to exclusively capture osteoporosis-associated pelvic fractures and minimize inclusion of fractures resulting from high-energy trauma or secondary causes of bone fragility. Patients admitted for nonoperative or operative management at a participating trauma center between January 2018 and September 2024 were included. Data were systematically entered into the German Pelvic Fracture Registry by trained personnel at participating centers using standardized case report forms based on medical charts, electronic health records, radiological reports, and discharge summaries. Data extraction for subsequent analysis took place on September 16, 2024. We initially selected patients aged ≥ 60 years to establish a geriatric cohort. This age threshold was chosen to focus on the typical osteoporotic population while excluding younger patients who might sustain low-energy fractures due to secondary causes of bone fragility such as metabolic bone disorders, malignancy, or chronic systemic diseases affecting bone metabolism. Since pelvic ring fractures represent the typical osteoporotic injury pattern in elderly patients and reflect a different phenotype compared to acetabular fractures, we systematically excluded all acetabular fractures from our cohort. Inclusion was restricted to patients whose injuries were classified according to the FFP classification, which was specifically developed for osteoporosis-associated pelvic ring fractures in elderly patients [34]. All patients without documented FFP classification were systematically excluded. The German Pelvic Fracture Registry categorizes pelvic ring injuries into isolated injuries, complex injuries, and polytrauma. We systematically excluded all patients classified as complex injuries or polytrauma to further ensure exclusion of high-energy mechanisms. Complex trauma was defined as pelvic ring injuries with significant soft tissue damage, vascular injuries, or neurological complications requiring intensive care management. Polytrauma patients were not present in our primary cohort. Finally, we excluded all patients for whom valid information regarding osteoporosis-specific pharmacotherapy before and after the fracture was not available in the registry. We systematically extracted data from the registry to identify patients with documented preexisting osteoporosis diagnoses prior to their pelvic ring fracture. This information was based on medical records entered. It is not recorded in the registry whether systematic osteoporosis diagnostics in the participating centers were performed.

**Fig. 1:**
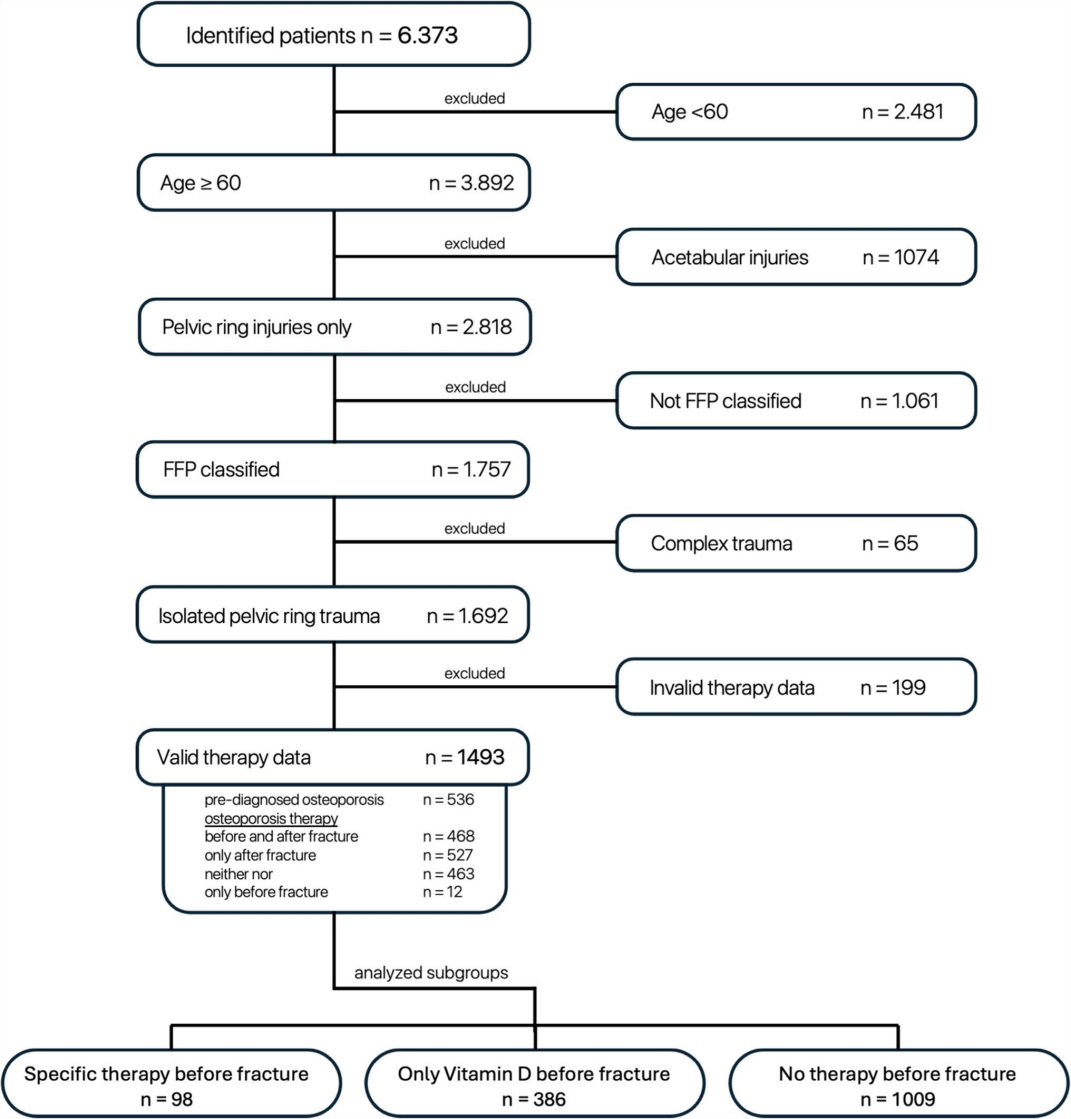
Flow diagram of study cohort selection. Systematic patient selection process for the fragility fracture of the pelvis (FFP) study cohort. Inclusion criteria comprised patients aged ≥ 60 years with FFP classified according to established criteria, occurring under low-energy trauma conditions between January 2018 and September 2024. Exclusion criteria eliminated high-energy trauma, pathological fractures, incomplete treatment documentation, major concomitant trauma (ISS > 16), active malignancy, and metabolic bone diseases other than osteoporosis. Sequential application of these criteria yielded a final cohort of 1,493 patients suitable for osteoporosis treatment pattern analysis

This systematic selection process resulted in a final cohort of 1493 patients. These patients were subsequently stratified into three treatment groups based on their osteoporosis management: specific osteoporosis medication (SOM; e.g. bisphosphonates and denosumab), vitamin D supplementation only (VitD), and no osteoporosis treatment (NOT), as summarized in Fig. 1.

**Fig. 2:**
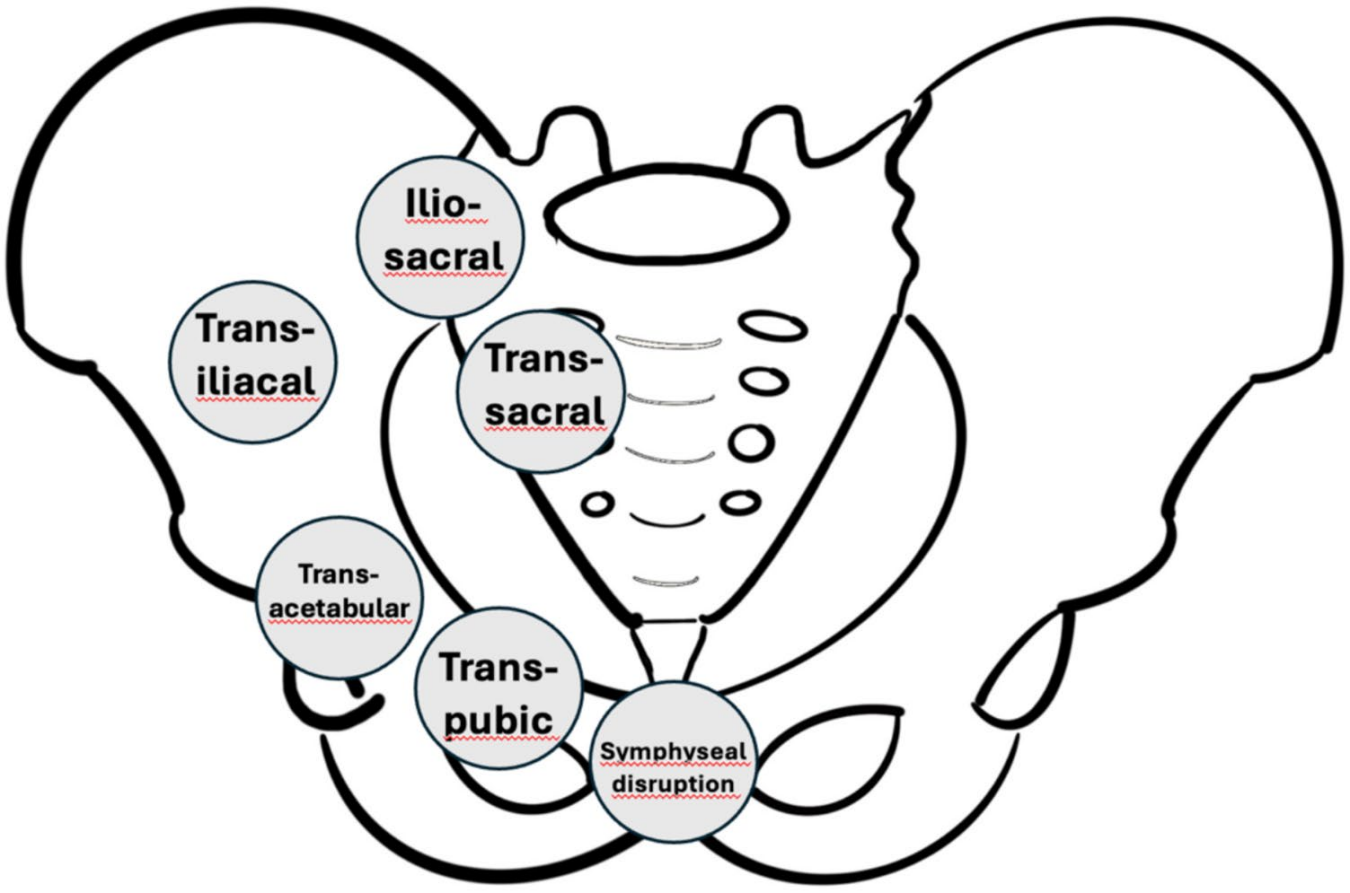
Analyzed fracture regions. Schematic illustration of analyzed pelvic ring fracture regions in the study cohort. Six distinct anatomical zones were evaluated: transsymphyseal (involving the pubic symphysis), transpubic (through the pubic rami), transacetabular (acetabular involvement), transiliac (iliac wing fractures), transiliosacral (sacroiliac joint disruption), and transsacral (sacral body fractures)

Analyzed parameters are summarized in Table 1. Study variables include patient characteristics such as sex, age, injury severity score (ISS), and information about the physical status according to the American Society of Anesthesiologists Physical Status Classification (ASA). Additionally, osteoporosis diagnosis and treatment status before and after fragility fracture of the pelvis (FFP), as well as fracture characteristics like FFP classification, posterior injury patterns, and dislocation, were taken into account. Besides, the analysis of the FFP classification anatomical fracture regions (Fig. 2) was investigated.

**Table 1:** Analyzed parameters and baseline characteristics

Category	Variables
Demographics osteoporosis	Sex Age ISS (injury severity score) ASA (American Society of Anesthesiologists Physical Status Classification) Life situation before fracture Discharge situation Known osteoporosis Treatment before and after FFP (fragility fractures of the pelvis) Treatment change
Fracture morphology	FFP classification system Posterior injury pattern Dislocation Fracture region (transsymphyseal, transpubic, transacetabular, transiliac, transiliosacral, transsacral)

Treatment modalities were categorized as nonoperative or operative treatment.

### Statistics

Statistical analysis was conducted using R (The R Foundation for Statistical Computing, Vienna). Results were expressed as absolute numbers, percentages, means, and standard deviation for normal Gaussian distributed data, medians and IQR for not-Gaussian distributed data. We performed the Kruskal–Wallis test for metric data and Chi-squared or Fisher tests for categorical data. A *p*-value < 0.05 was considered statistically significant.

## Results

The SOM group was statistically significantly older and included significantly more females compared to the VitD and NOT cohort (Table 2). Sex distribution differed significantly across the three treatment groups (*p* < 0.05, Table 2). Women comprised 94.9% of patients receiving specific osteoporosis medication (SOM), 89.9% of those receiving vitamin D supplementation only, and 79.9% of untreated patients (NOT). This demonstrates that women were overrepresented among treated patients, particularly in the SOM group, while men were disproportionately represented in the untreated cohort.

**Table 2:** Demographic characteristics, injury severity index (ISS), American Society of Anesthesiologists Physical Status Classification (ASA), life situation before hospital admission and discharge destination by osteoporosis therapy group. *p*-values for categorical variables were calculated using Chi-square test or Fisher’s exact test as appropriate, comparing proportions across the three treatment groups. *p*-values for continuous variables were calculated using ANOVA or Kruskal–Wallis test. Significant differences are indicated by *p* < 0.05

	SOM *n* 98 (6,6% of total)	VitD *n* 386 (25,9% of total)	NOT *n* 1009 (67,6% of total)	Total *n* 1493	*p*-value
Sex (f:m)	93:5 (94.1:5.1%)	347:39 (89.9:10.1%)	806:203 (79.9:20.1%)	1246:247 (83.5:16.5%)	< 0.05
Mean Age (y)	83.02 ± 7.87	83.09 ± 7.69	81,99 ± 8.19	82.34 ± 8.06	< 0.05
Mean ISS	9.55 ± 7.11	9.23 ± 5.78	10.58 ± 7.14	10.21 ± 6.88	Not significant
ASA					
I	2 (2%)	20 (5.2%)	73 (7.2%)	95 (6.4%)	< 0.05
II	30 (30.6%)	123 (31.9%)	378 (37.5%)	531 (35.6%)	< 0.05
III	40 (40.8%)	173 (44.8%)	379 (37.6%)	592 (39.7%)	< 0.05
IV	2 (2%)	3 (0.8%)	14 (1.4%)	19 (1.3%)	< 0.05
Life situation at admission					
At home, independent	70 (71.4%)	226 (58.6%)	670 (66.4%)	966 (64.7%)	< 0.05
At home, requiring care	17 (17.3%)	107 (27.7%)	183 (18.1%)	307 (20.6%)	< 0.05
Nursing home, independent	3 (3.1%)	15 (3.9%)	33 (3.3%)	51 (3.4%)	< 0.05
Nursing home, requiring care	7 (7.1%)	35 (9.1%)	98 (9.7%)	140 (9.4%)	< 0.05
Discharge situation					
At home	42 (42.9%)	155 (40.2%)	450 (44.6%)	647 (43.3%)	Not significant
Nursing home	17 (17.4%)	54 (14%)	160 (15.9%)	231 (15.5%)	Not significant
Rehabilitation	18 (18.4%)	91 (23.6%)	190 (18.8%)	299 (20%)	Not significant
Other	18 (18.4%)	72 (18.7%)	165 (16.4%)	255 (17.1%)	Not significant
Death	1 (1%)	10 (2.6%)	38 (3.8%)	49 (3.3%)	Not significant

Prior to a FFP, in the SOM cohort significantly more patients lived independently at home compared to the other groups. After a FFP, no statistically significant differences were observed between the groups regarding their living status.

### Pharmacological osteoporosis treatment before and after an FFP

Within the study population, 536 patients (38.5%) were diagnosed with osteoporosis.

Before an FFP, patients were distributed as followed: *n* = 98 (6.56%) SOM; *n* = 386 (25.85%) VitD; and *n *= 1009 (67.58%) NOT.

After an FFP 463 patients (31.5%) did not receive either SOM or VitD, 468 patients (31.8%) had no treatment change receiving SOM or VitD as before the FFP, in 527 patients (35.9%) SOM or VitD was started. In 12 patients (0.8%), SOM or VitD was stopped after the FFP.

**Fig. 3:**
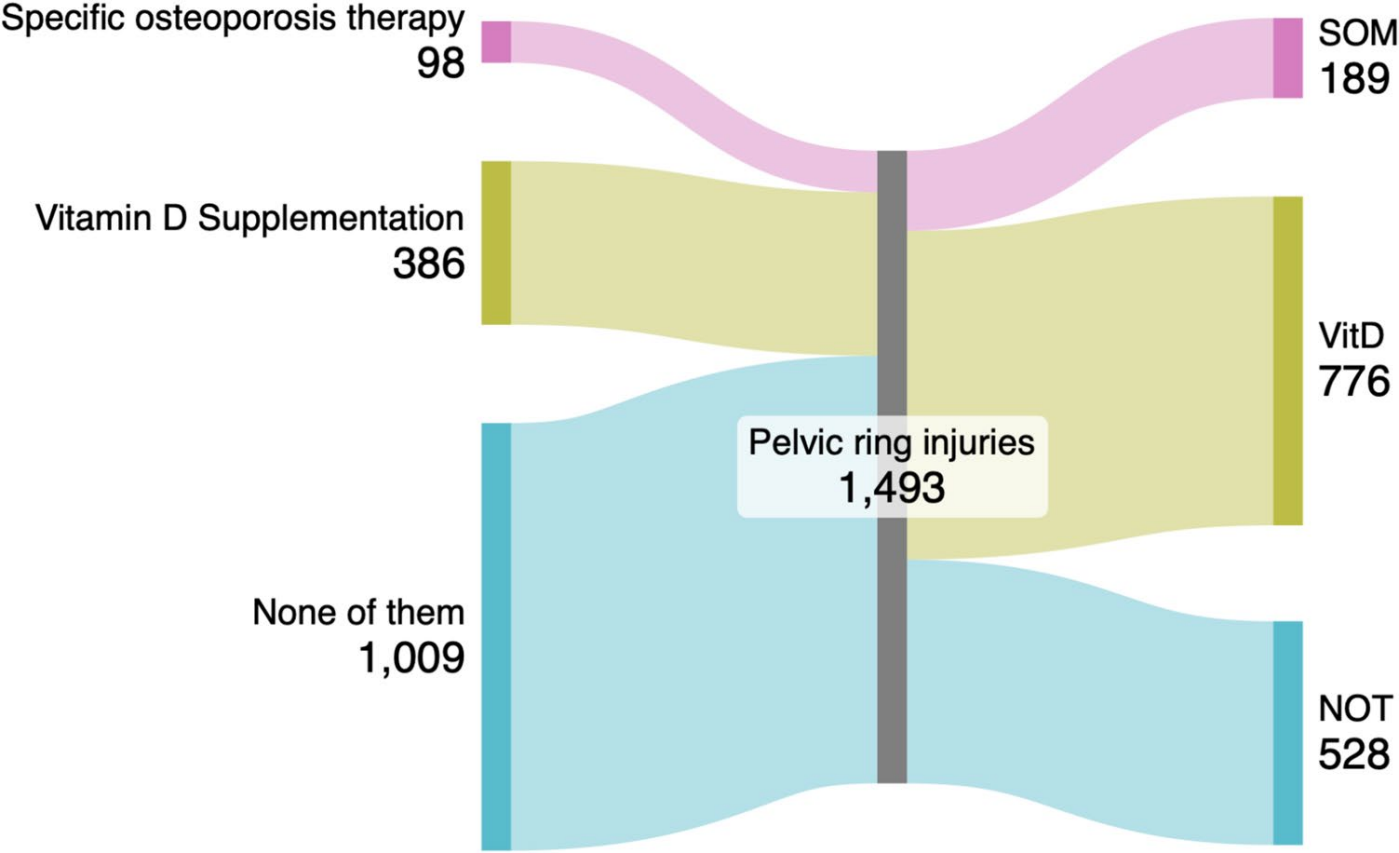
Group changes following a FFP. The left side of the figure presents the initial therapeutic groups: SOM, VitD and NOT. The right side, following fracture diagnosis and subsequent treatment, depicts the shift in this distribution. An almost twofold rise in the VitD is present, alongside of patients undergoing SOM. Illustrating therapeutic group transitions from pre-fracture to post-fracture osteoporosis management. The left panel displays initial treatment distribution: specific osteoporosis pharmacotherapy (SOM, n = 98), vitamin D supplementation (VitD, n = 386), and no treatment (NOT, n = 1009). The right panel demonstrates post-fracture therapeutic allocation following clinical intervention. Flow arrows indicate patient migration between treatment categories, revealing a predominant shift from untreated status toward vitamin D supplementation and specific pharmacotherapy initiation

Group changes following a FFP are highlighted in Fig. 3.

**Fig. 4:**
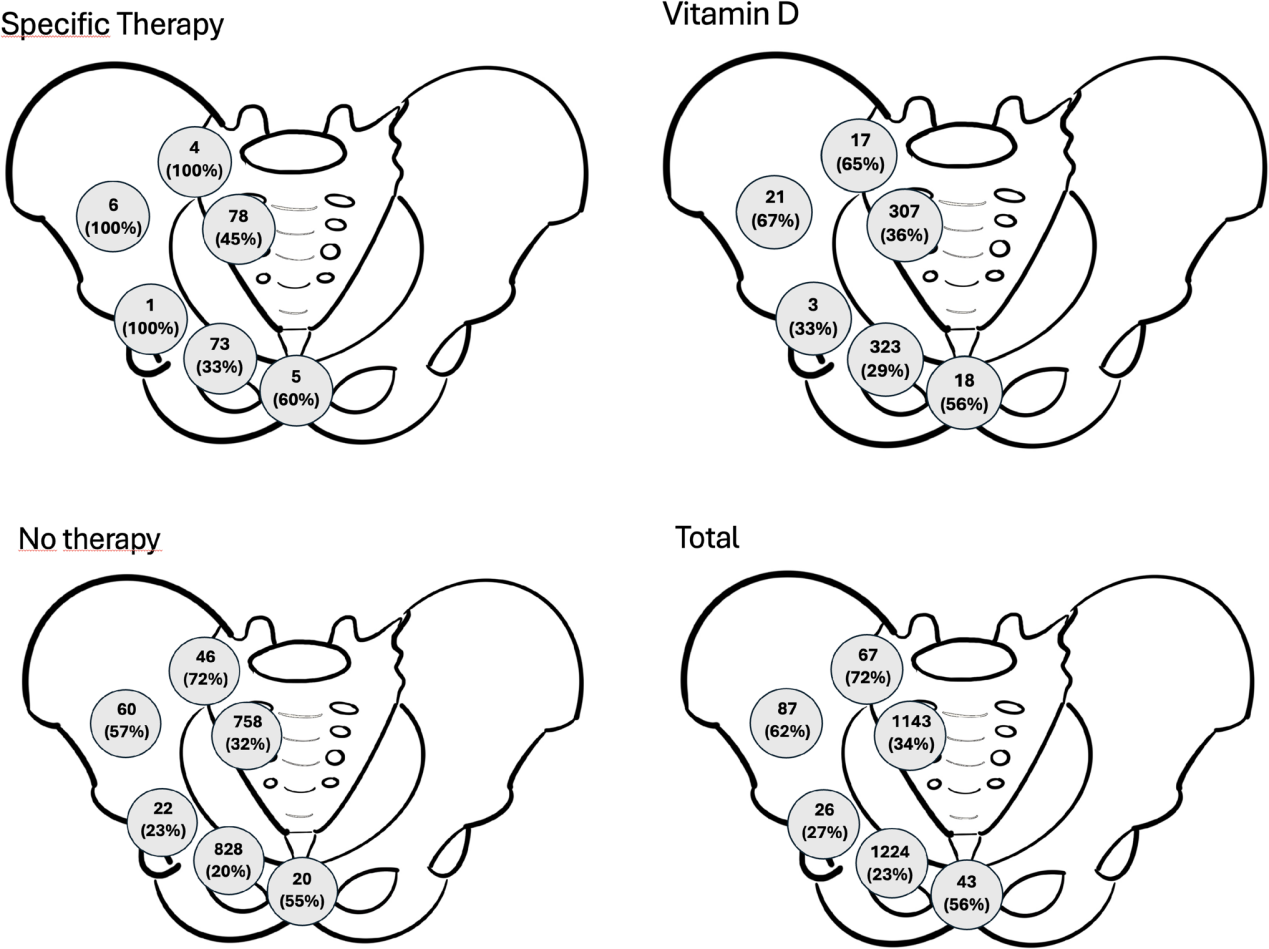
Total number of injuries at a specific location on the pelvic ring and percentage of dislocated fractures in that specific area and subgroup. Comparative analysis of fracture displacement patterns across anatomical regions stratified by osteoporosis treatment groups. Data represent the proportion of displaced fractures within each specific anatomical zone (transsymphyseal, transpubic, transacetabular, transiliac, transiliosacral, and transsacral) for patients receiving specific osteoporosis pharmacotherapy (SOM), vitamin D supplementation (VitD), or no treatment (NOT). Percentages are calculated relative to the total number of fractures occurring in each respective anatomical region within treatment subgroups

In summary, patients from the NOT group suffering a FFP primarily changed to the VitD group, and patients of the VitD group changed to the SOM group.

### Fracture morphology and treatment

Patients of the SOM group had more frequent FFP IV injuries and were more frequently operatively treated (Table 3). Transiliosacral and transacral injuries occurred similarly between the three groups.

**Table 3:** FFP classification distribution and incidence of dorsal injuries as well as operative stabilization

FFP classification	SOM *n* (% of 98)	VitD *n* (% of 386)	NOT n (% of 1009)	Total *n* (% of 1493)	*p*-value
**FFP I**	**13 (13.3)**	**58 (15.0)**	**208 (20.6)**	**279 (18.7)**	0.0064
FFP Ia	10 (10.2)	55 (14.2)	191 (18.9)	256 (17.1)	0.0036
FFP Ib	3 (3.1)	3 (0.8)	17 (1.7)	23 (1.5)	0.0036
**FFP II**	**43 (43.9)**	**200 (51.8)**	**526 (52.1)**	**769 (51.5)**	0.0064
FFP IIa	6 (6.1)	20 (5.2)	58 (5.7)	84 (5.6)	0.0036
FFP IIb	30 (30.6)	116 (30.1)	331 (32.8)	477 (31.9)	0.0036
FFP IIc	7 (7.1)	64 (16.6)	137 (13.6)	208 (13.9)	0.0036
**FFP III**	**9 (9.2)**	**38 (9.8)**	**75 (7.4)**	**122 (8.2)**	0.0064
FFP IIIa	5 (5.1)	11 (2.8)	19 (1.9)	35 (2.3)	0.0036
FFP IIIb	0	6 (1.6)	16 (1.6)	22 (1.5)	0.0036
FFP IIIc	4 (4.1)	21 (5.4)	40 (4.0)	65 (4.4)	0.0036
**FFP IV**	**33 (33.7)**	**90 (23.3)**	**200 (19.8)**	**323 (21.6)**	0.0064
FFP IVa	4 (4.1)	9 (2.3)	8 (0.8)	21 (1.4)	0.0036
FFP IVb	22 (22.4)	58 (15.0)	153 (15.2)	233 (15.6)	0.0036
FFP IVc	7 (7.1)	23 (6.0)	39 (3.9)	69 (4.6)	0.0036
**Dorsal injuries**					
Transiliosacral and transsacral injuries	82 (83.7)	324 (83.9)	804 (79.7)	1210 (81)	1
Operative treatment	45 (45.9)	116 (30.4)	291 (29.2)	452 (30.6)	0.0055

Patients of the SOM group had significantly more transpubic and transsacral fractures (Fig. 2, Supplemental 1).

## Discussion

The present registry-based analysis provides descriptive evidence regarding osteoporosis pharmacotherapy patterns and fracture characteristics in patients with fragility fractures of the pelvis. Five principal observations emerge from this investigation:

- A low prevalence of pre-fracture osteoporosis treatment.
- Patients receiving osteoporosis-specific medication demonstrated better prefracture functional status, with a higher proportion living independently at home prior to their fracture.
- Patients with preexisting specific osteoporosis therapy exhibited higher-instability FFP patterns (FFP III-IV) and underwent surgical intervention more frequently than untreated patients.
- Patients with prior specific osteoporosis medication demonstrated a higher prevalence of transpubic and transsacral fracture patterns with greater degrees of displacement.
- During hospitalization, osteoporosis-specific medication was initiated in 6.1% of all registered patients, increasing the total proportion receiving SOM at discharge to 12.7%. Vitamin D supplementation demonstrated more substantial uptake, with prescriptions rising from 25.9% on admission to 52.0% at discharge.

Given the retrospective, observational nature of this registry analysis, these findings represent descriptive observations rather than evidence of causal relationships. The observed differences between treatment groups may reflect confounding by indication (patients receiving treatment may have had more severe underlying osteoporosis), selection bias, or other unmeasured factors. We cannot determine from these data whether treatment influences fracture characteristics or whether fracture patterns reflect disease severity that prompted treatment initiation.

### Prefracture osteoporosis management

The observed treatment rate of 32% prior to FFP corresponds with contemporary literature documenting inadequate osteoporosis management in at-risk populations. Smith et al. reported comparable rates (27.1%) among 947 patients with pelvic fragility fractures, with 96% having never undergone formal osteoporosis evaluation [23]. McCloskey et al. documented that approximately 75% of elderly women at elevated fracture risk remained untreated, while Singer et al. found treatment rates of 14.1% among postmenopausal women with fragility fractures, though these latter studies were not specific to pelvic fractures [24, 25].

This treatment gap reflects multiple systemic and patient-specific factors and poses a huge socioeconomic and healthcare problem. Insufficient screening protocols, limited access to bone densitometry, and inadequate referral pathways could contribute to underdiagnosis. Patient-related factors including medication concerns, cost considerations, and perceived treatment necessity could further influence therapeutic initiation and adherence.

In 2019, Germany recorded approximately 5.66 million individuals with osteoporosis (6.1% of the population) and 831,000 annual fragility fractures, with projections indicating 928,000 fractures by 2025—a 28% increase since 2010 [35, 36]. Pelvic ring injuries (Fig. 4) specifically affected 40,571 hospitalized patients in 2019, with incidence rates increasing 39% over the preceding decade. Healthcare costs reached €13.8 billion in 2019 (3.7% of national health expenditure), with nonhip fractures including pelvic injuries accounting for 42% of total fracture costs [35–37]. Despite robust evidence demonstrating fracture risk reduction of 20–68% with a specific osteoporosis medication, a substantial treatment gap persists, with 76% of high-risk women remaining inadequately treated [35, 38]. This represents a critical missed opportunity, as treating 1000 patients over three years prevents 100 osteoporotic fractures while causing only 1.3 atypical femoral fractures [39]. Systematic closure of this treatment gap could prevent hundreds of thousands of osteoporosis-associated fractures annually, mitigating projected cost increases and generating billions of euros in healthcare savings while substantially reducing patient morbidity and mortality associated with fragility fractures.

#### Functional status and treatment history

Patients receiving SOM demonstrated higher rates of independent living prior to fracture (71%) compared to those receiving VitD (59%) or NOT (66%). This observation may indicate that osteoporosis treatment serves as a marker for comprehensive healthcare engagement rather than a direct causative factor for functional independence.

While direct evidence linking prefracture osteoporosis treatment to functional status remains limited, indirect evidence supports this association. Marrinan et al. identified age and acute medical conditions as determinants of postfracture outcomes in 110 elderly patients with pelvic fractures [26]. In a prospective study of 520 elderly patients with hip fractures, Makridis et al. reported that 15% had received osteoporosis treatment prior to fracture occurrence [27]. Patients who had undergone prefracture osteoporotic therapy demonstrated statistically significant improvements in functional recovery, reduced refracture rates, and enhanced quality-of-life scores as measured by the EQ-5D instrument during the approximately 2-year postoperative follow-up period compared to untreated patients [27]. These findings underscore the clinical relevance of timely osteoporotic intervention in this patient population.

#### Fracture severity paradox

A notable finding concerns the increased prevalence of FFP type IV fractures (33.7%) and surgical intervention requirements (45.9%) among patients receiving SOM. Thus, treated patients sustain more severe fractures.

Patients prescribed SOM likely represent individuals with advanced osteoporotic disease or elevated baseline fracture risk [2], as specific osteoporotic medication was presumably reserved for those with severe osteoporosis, introducing considerable selection bias. Comprehensive epidemiological studies are needed to address this limitation. The persistence of severe fracture patterns despite treatment may reflect inadequate treatment duration, suboptimal adherence, or refractory disease requiring alternative therapeutic approaches.

In addition, antiresorptive therapies, while reducing overall fracture incidence, may alter bone remodeling patterns and mechanical properties [28]. This phenomenon parallels observations of atypical femoral fractures associated with prolonged bisphosphonate therapy [22]. Similar mechanisms might influence pelvic fracture configurations, potentially predisposing to complex injury patterns when sufficient force is applied.

Yet, an association between fracture displacement and specific osteoporotic medication appears plausible, given the current treatment gap of 76% in Germany [35, 38]. This gap implies a high prevalence of advanced osteoporotic disease within both the vitamin D supplementation and untreated cohorts, which would theoretically present with increased displacement across fracture classifications. However, this association is not observed in any FFP subtype.

Furthermore, stable fracture patterns in treated patients might heal without hospitalization or remain clinically occult under nonoperative management including continued SOM. This could result in the selective hospitalization of patients with unstable fracture patterns requiring surgical intervention.

Moreover, a subset of patients may maintain compromised bone microarchitecture despite appropriate pharmacotherapy. These individuals might experience fewer total fractures due to partial therapeutic benefit while sustaining more severe injuries when trauma occurs.

The smaller absolute number of patients in the SOM cohort, despite representing a theoretically higher risk population, suggests therapeutic efficacy in preventing fractures overall. This aligns with established evidence demonstrating fracture risk reduction with bisphosphonates, denosumab, and teriparatide [20, 21].

#### Surgical implications

The increased operative intervention rate in the SOM group correlates with fracture displacement, complexity, and a higher FFP classification.

Furthermore, current evidence supports bilateral stabilization of the posterior pelvic ring, even in unilateral injuries, as biomechanical studies demonstrate enhanced multidimensional stability of both the anterior and posterior pelvic ring components with this approach [30]. Additionally, the risk of contralateral fracture progression in osteoporotic patients with initially unilateral injuries provides compelling justification for prophylactic bilateral fixation strategies [31].

#### Treatment initiation during hospitalization

SOM or VitD was initiated during hospitalization in approximately 50% of previously untreated FFP patients. This finding aligns with Gregory et al., who reported 35% treatment initiation rates among 191 hip fracture patients during acute hospitalization [29]. Smith et al. documented lower rates, with only 4% of treatment-naive pelvic fracture patients beginning therapy within one year [23].

These data highlight persistent gaps in secondary fracture prevention despite the established benefits of post-fracture osteoporosis treatment [32]. Factors influencing treatment initiation include discharge diagnosis documentation, treating service specialty, and care coordination between hospital and community providers. Implementation of fracture liaison services and standardized treatment protocols may improve initiation rates [32, 33].

#### Study limitations

To our knowledge, this represents the first observational study examining the relationship between osteoporosis, osteoporotic treatment, and the demographic characteristics, fracture classification, and postfracture osteoporosis therapy in patients with FFP. Several important limitations must be acknowledged when interpreting our findings. First and most importantly, the retrospective, observational nature of this registry-based study precludes causal inference. The observed differences in fracture patterns between treatment groups may reflect confounding by indication rather than treatment effects. Patients receiving specific osteoporosis medication likely had more severe underlying osteoporosis, previous fragility fractures, or other risk factors that prompted treatment initiation. These same factors could independently increase the risk of more severe or displaced fractures, regardless of treatment. Without randomization or multivariable adjustment for baseline osteoporosis severity, previous fracture history, comorbidities, and other confounders, we cannot determine whether the observed patterns represent true treatment-related phenomena or simply reflect differences in baseline risk profiles.

Second, the German Pelvic Fracture Registry, while comprehensive in capturing fracture characteristics and acute treatment patterns, does not systematically collect detailed osteoporosis pharmacotherapy data including treatment duration, cumulative medication exposure, adherence patterns, or specific formulations. Other important confounders including bone mineral density measurements and biochemical markers were not available for analysis. All factors that could influence fracture risk and morphology. Third, we cannot determine the temporal relationship between treatment initiation and fracture occurrence with sufficient precision to establish dose–response relationships.

Furthermore, immobilization duration is clinically relevant as prolonged bed rest in elderly patients with fragility fractures increases the risk of complications such as thromboembolism, pneumonia, sarcopenia, and functional decline and may differ between operative and nonoperative management approaches. Our finding that patients receiving specific osteoporosis medication demonstrated higher rates of FFP IV fractures and greater fracture displacement warrants careful consideration. Black et al. demonstrated that prolonged bisphosphonate use increases the risk of atypical femur fractures [22], characterized by distinct morphological features reflecting altered bone remodeling. Given that both femoral and pelvic bones are weight-bearing structures potentially susceptible to similar stress-related changes under long-term antiresorptive therapy, we considered whether our findings might represent a parallel phenomenon in the pelvis.

The registry data do not provide sufficient morphological detail to determine whether fractures in the SOM group exhibit truly distinct features analogous to the well-defined cortical breaking, transverse fracture lines, and periosteal reactions characteristic of atypical femur fractures. The observational nature of our study precludes determination of whether the association reflects a causal effect of treatment or confounding by indication. Patients receiving SOM may have had more severe underlying osteoporosis, placing them at higher risk for severe fractures regardless of treatment. Therefore, while the combination of increased fracture severity and displacement in treated patients is noteworthy and merits further investigation, we cannot conclude that this represents a distinct “atypical pelvic fracture” entity. Rather, our observations may reflect either more severe manifestations of typical fragility fractures in patients with advanced osteoporosis, the effects of confounding by indication, or potentially subtle alterations in bone structure not adequately captured by current classification systems. Future prospective studies with detailed radiographic analysis, histomorphometry, and comprehensive treatment history would be necessary to definitively address this question.

Further limitations are the relatively small SOM cohort size, which limits statistical power, though this may reflect therapeutic success in fracture prevention.

Functional outcomes and long-term complications were not evaluated, limiting assessment of treatment impact beyond the acute hospitalization period. The single healthcare system setting may limit generalizability to other populations and care delivery models.

## Conclusions

This analysis demonstrates that while specific osteoporosis pharmacotherapy appears to reduce overall FFP incidence, breakthrough fractures in treated patients may present with increased severity and complexity requiring surgical intervention. These findings underscore the complexity of osteoporosis management in the context of pelvic fragility fractures. The observation that treated patients may sustain more severe fractures when injuries occur emphasizes the importance of comprehensive fracture prevention strategies extending beyond pharmacological intervention. Fall prevention programs, balance training, and environmental modifications remain essential components of fracture risk reduction.

The apparent paradox of increased fracture severity in treated patients should not discourage osteoporosis therapy initiation but rather inform treatment expectations and monitoring strategies. Regular assessment of treatment response through bone turnover markers and densitometry may identify patients requiring therapeutic modification. However, the low prefracture treatment rates and suboptimal therapy initiation during hospitalization highlight persistent gaps in osteoporosis care delivery. These findings emphasize the need for systematic approaches to osteoporosis screening, treatment initiation, and comprehensive fracture prevention strategies in at-risk populations.

Future epidemiologic evaluations and prospective studies incorporating detailed treatment histories, adherence data, and long-term functional outcomes are warranted to further elucidate the relationship between osteoporosis therapy and pelvic fracture patterns and to prevent pathologic fractures and reduce socioeconomic costs.

## Data Availability

Data are available on reasonable request by the senior author.
